# Supplementary material for: Association between dental caries and Helicobacter pylori infection in Japanese adults: A cross-sectional study
Source: PLoS One. 2022 Jul 14;17(7):e0271459. doi: 10.1371/journal.pone.0271459 (PMC9282579; doi:10.1371/journal.pone.0271459)
Supplement: Attachment — Submitted filename: PONE-D-21-36232.pdf [file pone.0271459.s001.pdf]

PLOS ONE

Association between dental caries and Helicobacter pylori infection in Japanese adults: A cross-sectional study

--Manuscript Draft--

Manuscript Number:	PONE-D-21-36232
Article Type:	Research Article
Full Title:	Association between dental caries and Helicobacter pylori infection in Japanese adults: A cross-sectional study
Short Title:	Association between dental caries and Helicobacter pylori infection in Japanese adults
Corresponding Author:	Takaaki Tomofuji<br>Asahi University: Asahi Daigaku<br>Hizuho, JAPAN
Keywords:	Helicobacter Pylori; Helicobacter Infections; Dental Caries; Cross-Sectional Study
Abstract:	Helicobacter pylori (H. pylori) is widely known as a cause of gastric disorders. Presence of H. pylori in dental pulp has been reported. Dental caries may influence the presence or absence of systemic H. pylori infection by serving as a source of H. pylori . In this cross-sectional study, we examined whether H. pylori infection in blood were associated with dental caries in Japanese adults. The participants were 752 individuals (513 males and 239 females, mean age 53.8 years) who underwent both H. pylori testing (H. pylori antibody test and pepsinogen test) and dental checkups at the Asahi University Hospital Human Health Center in fiscal years 2018 and 2019. Those diagnosed as positive for H. pylori antibody test or positive for serum pepsinogen test as H. pylori test in the human health checkup were judged as those with H. pylori infection in the blood. In our study, 83 participants (11%) were determined to be infected with H. pylori in the blood. The percentage of those with decayed teeth was higher in participants with H. pylori infection in blood than in those without H. pylori infection in blood (p< 0.001). The logistic analysis showed that presence of H. pylori infection in blood was positively associated with those with decayed teeth (OR, 5.656; 95% CI, 3.374 to 9.479) after adjusting for age, gender, gastric disease, regular dental checkups, antibiotic medication history, and decayed teeth. Furthermore, the prevalence rate of H. pylori infection in blood increased according to number of decayed teeth (p< 0.001). The results indicate that H. pylori infection in blood were associated with decayed teeth. Untreated dental caries may have an impact on systemic H. pylori infection.
Order of Authors:	Komei Iwai
	Tetsuji Azuma
	Takatoshi Yonenaga
	Kazutoshi Watanabe
	Akihiro Obora
	Fumiko Deguchi
	Takao Kojima
	Takaaki Tomofuji
Additional Information:	
Question	Response

Powered by Editorial Manager® and ProduXion Manager® from Aries Systems Corporation

Financial Disclosure<br><br>Enter a financial disclosure statement that describes the sources of funding for the work included in this submission. Review the submission guidelines for detailed requirements. View published research articles from PLOS ONE for specific examples.<br><br>This statement is required for submission and will appear in the published article if the submission is accepted. Please make sure it is accurate.<br><br>Unfunded studies<br>Enter: The author(s) received no specific funding for this work.<br><br>Funded studies<br>Enter a statement with the following details:<br>• Initials of the authors who received each award<br>• Grant numbers awarded to each author<br>• The full name of each funder<br>• URL of each funder website<br>• Did the sponsors or funders play any role in the study design, data collection and analysis, decision to publish, or preparation of the manuscript?<br>• NO - Include this sentence at the end of your statement: The funders had no role in study design, data collection and analysis, decision to publish, or preparation of the manuscript.<br>• YES - Specify the role(s) played.<br><br>* typeset	NO

Competing Interests<br><br>Use the instructions below to enter a competing interest statement for this submission. On behalf of all authors, disclose any competing interests that could be perceived to bias this work—acknowledging all financial support and any other relevant financial or nonfinancial competing interests.<br><br>This statement is required for submission and will appear in the published article if the submission is accepted. Please make sure it is accurate and that any funding sources listed in your Funding Information later in the submission form are also declared in your Financial Disclosure	The authors have declared that no competing interests exist.
statement.<br><br>View published research articles from PLOS ONE for specific examples.<br><br>NO authors have competing interests<br><br>Enter: The authors have declared that no competing interests exist.<br><br>Authors with competing interests<br><br>Enter competing interest details beginning with this statement:<br><br>I have read the journal's policy and the authors of this manuscript have the following competing interests: [insert competing interests here]<br><br>* typeset	

Powered by Editorial Manager® and ProduXion Manager® from Aries Systems Corporation

Ethics Statement<br><br>Enter an ethics statement for this submission. This statement is required if the study involved:<br><br>• Human participants<br>• Human specimens or tissue<br>• Vertebrate animals or cephalopods<br>• Vertebrate embryos or tissues<br>• Field research<br><br>Write "N/A" if the submission does not require an ethics statement.<br><br>General guidance is provided below. Consult the submission guidelines for detailed instructions. Make sure that all information entered here is included in the Methods section of the manuscript.	Our study was approved by the Ethics Committee of Asahi University (No. 27010), and was performed in accordance with the Declaration of Helsinki. All residents who participated provided written informed consent.

Powered by Editorial Manager® and ProduXion Manager® from Aries Systems Corporation

Format for specific study types<br><br>Human Subject Research (involving human participants and/or tissue)<br>• Give the name of the institutional review board or ethics committee that approved the study<br>• Include the approval number and/or a statement indicating approval of this research<br>• Indicate the form of consent obtained (written/oral) or the reason that consent was not obtained (e.g. the data were analyzed anonymously)<br><br>Animal Research (involving vertebrate animals, embryos or tissues)<br>• Provide the name of the Institutional Animal Care and Use Committee (IACUC) or other relevant ethics board that reviewed the study protocol, and indicate whether they approved this research or granted a formal waiver of ethical approval<br>• Include an approval number if one was obtained<br>• If the study involved non-human primates, add additional details about animal welfare and steps taken to ameliorate suffering<br>• If anesthesia, euthanasia, or any kind of animal sacrifice is part of the study, include briefly which substances and/or methods were applied<br><br>Field Research<br><br>Include the following details if this study involves the collection of plant, animal, or other materials from a natural setting:<br>• Field permit number<br>• Name of the institution or relevant body that granted permission	
Data Availability<br><br>Authors are required to make all data underlying the findings described fully available, without restriction, and from the time of publication. PLOS allows rare exceptions to address legal and ethical concerns. See the PLOS Data Policy and FAQ for detailed information.	No - some restrictions will apply

Powered by Editorial Manager® and ProduXion Manager® from Aries Systems Corporation

A Data Availability Statement describing where the data can be found is required at submission. Your answers to this question constitute the Data Availability Statement and will be published in the article, if accepted.<br><br>Important: Stating ‘data available on request from the author’ is not sufficient. If your data are only available upon request, select ‘No’ for the first question and explain your exceptional situation in the text box.<br><br>Do the authors confirm that all data underlying the findings described in their manuscript are fully available without restriction?	

Describe where the data may be found in full sentences. If you are copying our sample text, replace any instances of XXX with the appropriate details.<br><br>• If the data are held or will be held in a public repository, include URLs, accession numbers or DOIs. If this information will only be available after acceptance, indicate this by ticking the box below. For example: All XXX files are available from the XXX database (accession number(s) XXX, XXX.).<br>• If the data are all contained within the manuscript and/or Supporting Information files, enter the following: All relevant data are within the manuscript and its Supporting Information files.<br>• If neither of these applies but you are able to provide details of access elsewhere, with or without limitations, please do so. For example:<br><br>Data cannot be shared publicly because of [XXX]. Data are available from the XXX Institutional Data Access / Ethics Committee (contact via XXX) for researchers who meet the criteria for access to confidential data.<br><br>The data underlying the results presented in the study are available from (include the name of the third party	Data cannot be shared publicly because of personal Information Protection. Data are available from the Ethics Committee of Asahi University (No. 27010) for researchers who meet the criteria for access to confidential data.

and contact information or URL).<br>• This text is appropriate if the data are owned by a third party and authors do not have permission to share the data.<br><br>* typeset	
Additional data availability information:	

Text

Click here to access/download;Manuscript;Manuscript, Table PLOS ONE.docx

Association between dental caries and *Helicobacter pylori* infection in Japanese adults: A cross-sectional study

Komei Iwai[1], Tetsuji Azuma[1], Takatoshi Yonenaga[1], Kazutoshi Watanabe[2], Akihiro Obora[2], Fumiko Deguchi[2], Takao Kojima[2], Takaaki Tomofuji[1※]

[1]Department of Community Oral Health, School of Dentistry, Asahi University, 1851 Hozumi, Mizuho, Gifu 501-0296, Japan.

[2] Asahi University Hospital, 3-23 Hashimoto-cho, Gifu, Gifu 500-8523, Japan.

Abstract

Helicobacter pylori (*H. pylori*) is widely known as a cause of gastric disorders. Presence of *H. pylori* in dental pulp has been reported. Dental caries may influence the presence or absence of systemic *H. pylori* infection by serving as a source of *H. pylori*. In this crosssectional study, we examined whether *H. pylori* infection in blood were associated with dental caries in Japanese adults. The participants were 752 individuals (513 males and 239 females, mean age 53.8 years) who underwent both *H. pylori* testing (*H. pylori* antibody test and pepsinogen test) and dental checkups at the Asahi University Hospital Human Health Center in fiscal years 2018 and 2019. Those diagnosed as positive for *H. pylori* antibody test or positive for serum pepsinogen test as *H. pylori* test in the human health checkup were judged as those with *H. pylori* infection in the blood. In our study, 83 participants (11%) were determined to be infected with *H. pylori* in the blood. The percentage of those with decayed teeth was higher in participants with *H. pylori* infection in blood than in those without *H. pylori* infection in blood ($p < 0.001$). The logistic analysis showed that presence of *H. pylori* infection in blood was positively associated with those with decayed teeth (OR, 5.656; 95% CI, 3.374 to 9.479) after adjusting for age, gender, gastric disease, regular dental checkups, antibiotic medication history, and decayed teeth. Furthermore, the prevalence rate of *H. pylori* infection in blood increased

according to number of decayed teeth ($p < 0.001$). The results indicate that *H. pylori* infection in blood were associated with decayed teeth. Untreated dental caries may have an impact on systemic *H. pylori* infection.

Corresponding author

Takaaki Tomofuji; tomofu@dent.asahi-u.ac.jp; Department of Community Oral Health, School of Dentistry, Asahi University, 1851 Hozumi, Mizuho, Gifu 501-0296, Japan.

Key Words

Helicobacter Pylori, *Helicobacter* Infections, Dental Caries, Cross-Sectional Study.

Introduction

Helicobacter pylori (*H. pylori*) is a Gram-negative spiral rod that causes gastric diseases such as chronic gastritis, gastric ulcer, duodenal ulcer, and gastric cancer [1, 2, 3]. *H. pylori* is an International Agency for Research on Cancer (IARC) Group 1 carcinogen [4] and the attributable fraction for gastric cancer is close to 90% [5]. The 2012 IARC report pointed out that more than half of the world's population is infected with *H. pylori* [4]. According to a 2012 report by Hirayama et al., the infection rate of *H. pylori* among Japanese was about 28% in a survey conducted between 2008 and 2011, and the infection rate decreased with decreasing age [6]. However, *H. pylori*-associated gastric cancer was the second most common cancer among men and the fourth most common among women in Japan in 2020, and it remains a disease that affects many people [7]. Therefore, prevention of *H. pylori* infection is a very important project in the field of public health in Japan.

In recent years, *H. pylori* has been detected in dental plaque, saliva, and pediatric dental pulp [8, 9, 10]. Our previous study also detected *H. pylori* in dental pulp and dental plaque of Japanese adults [11]. Using a nested PCR method based on the urea gene sequence [12], we have detected *H. pylori* in the dental pulp of 23 (12.0%) and in the dental plaque of 2 (1.0%) of 192 subjects. Furthermore, the detection of *H. pylori* in dental pulp was

significantly associated with gastric *H. pylori* infection [11]. It is feasible that *H. pylori* infection in dental pulp was associated with systemic *H. pylori* infection.

Dental caries is a disease in which oral bacteria cause parenchymal defects in the tooth structure due to the acid produced from carbohydrates, and it is the most common cause of pulp infection [13]. It is reported that bacteria infected with dental pulp can migrate directly to blood circulation and cause atherosclerotic properties in blood vessels [14]. Like this, *H. pylori* infected with dental pulp due to dental caries may be detrimental to the risk of systemic *H. pylori* infection. In other words, dental caries may increase the risk of systemic *H. pylori* infection. However, there is very little literature on the relationship between dental caries and systemic *H. pylori* infection. In the present work, we hypothesized that dental caries might be associated with the risk of systemic *H. pylori* infection. The blood antibody tests are widely used to confirm the presence of *H. pylori* infection in the whole body [15]. Therefore, the aim of this cross-sectional study was to investigate the relationship between dental caries and *H. pylori* infection in blood in Japanese adults.

Material and Methods

Participants

The participants were 782 participants who visited the Asahi University Hospital Human Health Center between April 2018 and March 2020 underwent both *H. pylori* testing (*H. pylori* antibody test and pepsinogen test) and dental checkups. All participants gave written informed consent. Of these, participants with unknown *H. pylori* test results (27 participants) due to incompleteness at the time of the blood test and participants with unknown caries in the dental checkup (3 participants) were excluded from the analysis. As a result, 752 participants (513 males and 239 females, mean age 53.8 years) were included in the final analysis.

Evaluation of the presence of *H. pylori* infection in the blood

Detection of *H. pylori* infection in the blood was done using *H. pylori* antibody test or pepsinogen test. In our study, those who tested positive for *H. pylori* antibody test or positive for serum pepsinogen test were determined to have *H. pylori* infection in their blood. This test has been reported to have excellent performance (sensitivity; 88-96%, specificity; 89-100%) [16].

Commented [HYE1]: Do both tests have the same degree of sensitivity? Please elaborate on this.

Oral examination

Four attending dentists checked the oral conditions, including number of decayed, missing teeth, and periodontal condition for each patient [17]. To evaluate periodontal condition, the periodontal probe (Hu-Friedy, USA) was used and the coded values of the Community Periodontal Index (CPI) were utilized [18]. The maximum value was classified into three code values (code 0 = periodontal pocket less than 3 mm, code 1 = periodontal pocket 4-5 mm, code 2 = periodontal pocket more than 6 mm), and codes 1 and 2 were evaluated as having periodontal disease.

Self-administered questionnaire

A self-administered questionnaire was used to investigate the participants' age, gender, smoking habits, drinking habits, medical history, sleep disorders, regular dental checkups, and antimicrobial medications. For smoking habits, those who smoked at least one cigarette per day were included (yes or no) [19]. For alcohol consumption, those who regularly drink alcohol at least once a week were included (yes or no) [20]. For regular dental checkups, those who regularly visit a dentist at least once six months were included (yes or no) [21].

Statistical analysis

Significant differences in the characteristics of the presence or absence of *H. pylori* infection in blood were assessed using the chi-square test and Mann-Whitney U test. Univariate and multivariate stepwise logistic regression analyses were performed with the presence of *H. pylori* in blood infection as the dependent variable. The third category of variables related to the sample (age, gender) and the other variables related to *H. pylori* infection in blood (gastric disease, regular dental checkups, antibiotic medication history, and decayed teeth), which were adjusted for in these analyses. Differences in the prevalence of *H. pylori* infection in blood among different percentage of those with decayed teeth were assessed using the chi-square test; variables with $p < 0.10$ were excluded from the model, and variables with $p < 0.05$ were included in the model. Independent variables with $p < 0.05$ were selected in univariate models. All data were analyzed using a statistical analysis software (SPSS statistics version 27; IBM Japan, Tokyo, Japan). All p-values < 0.05 were considered statistically significant.

Research ethics

Our study was approved by the Ethics Committee of Asahi University (No. 27010), and was performed in accordance with the Declaration of Helsinki. All residents who participated provided written informed consent. Our study is a cross-sectional study and follows the STROBE guidelines.

Results

Table 1 shows the characteristics of our study participants with and without *H. pylori* infection in blood. In our study, 83 participants (11%) were positive for *H. pylori* infection in blood. The participants with *H. pylori* infection in blood were characterized by significantly higher rates of those with decayed teeth (p = 0.001) compared to uninfected participants. The participants with *H. pylori* infection in blood were characterized by higher rates of regular dental checkups (p = 0.077) and were also lower antibiotic medication history (yes; p = 0.096) compared to uninfected participants, but this was not significant. The results of univariate logistic regression analysis with *H. pylori* infection in blood as the dependent variable are shown in Table 2. The results showed that the presence of *H. pylori* in blood infection was significant associated with those with decayed teeth (OR, 4.929; 95% CI, 2.998 to 8.114).

Table 3 shows the adjusted odds ratios and 95% CI for *H. pylori* infection in blood according to the analyzed factors in participants. The participants with *H. pylori* infection in blood were significantly associated with those with decayed teeth (OR, 5.017; 95% CI, 3.031 to 8.305) after adjusting for age and gender. After additional adjustments for gastric disease, regular dental checkups, antibiotic medication history, and decayed teeth, the

participants of *H. pylori* infection in blood were significantly associated with those with decayed teeth (OR, 5.656; 95% CI, 3.374 to 9.479).

Table 4 shows the difference in *H. pylori* infection in blood rates according to number of decayed teeth. *H. pylori* infection in blood rate among participants with one decayed tooth was 20% (13/66), among participants with two decayed teeth was 33% (10/30), and among participants with three or more decayed teeth was 63% (10/16), respectively. The infection rate for *H. pylori* tended to increase with the number of decayed teeth ($p < 0.001$).

Discussion

To the best of our knowledge, this was the first study to examine the association between dental caries and *H. pylori* infection in blood in Japanese adults. The results showed that the participants with *H. pylori* infection in blood had higher rates of those with decayed teeth than those without *H. pylori* infection in blood. The logistic regression analyses also revealed that presence of *H. pylori* infection in blood was associated with those with decayed teeth after adjusting age, gender, gastric disease, regular dental checkups, antibiotic medication history, and decayed teeth. Furthermore, the prevalence rate of *H. pylori* infection in blood increased according to the number of decayed teeth. These suggest that the risk of *H. pylori* infection in blood increases as the those with decayed teeth, and as the number of decayed teeth increases.

It has been reported that the caries cavity is difficult to reach with a brush during oral cleaning, making it difficult to remove accumulated oral bacteria [22]. Therefore, the caries cavity could serve as a reservoir for *H. pylori* because of self-cleaning is difficult to work, contributing to induce systemic *H. pylori* infection. Although further studies are needed, early treatment of decayed teeth may be beneficial to reduce the risk of systemic *H. pylori* infection.

A previous study has reported that people who harbor severe dental caries have a higher detection rate of *H. pylori* in their saliva than those who do not [23]. The other study of deciduous teeth of 4-7 years old children also reported that subjects with a higher number of cavities had a higher proportion of *H. pylori* infection [24]. These observations are consistent with the present study which showed a significant association between dental caries and *H. pylori* infection.

Commented [HYE2]: In your study, you used the number of decayed and missing teeth which was a quantitative enumeration that does not reflect the severity of dental caries. You may hint to that in the discussion part.

In our study, the prevalence rate of *H. pylori* infection in blood among all participants was 11%. This value was lower than previously reported prevalence rate of *H. pylori*infected subjects in Japan [6, 25]. This may be due to the fact that the average age of the participants in our study was 53.8 years, which was lower than in past studies.

In our study, we find no association between Periodontal pocket and *H. pylori* infection in blood. This observation is agreement with previous studies, which reported that there was no significant correlation between *H. pylori* infection and severity of periodontitis [11, 26]. However, a previous study also has reported that a higher proportion of *H. pylori*infected patients had periodontal disease compared to *H. pylori*-uninfected patients [27]. Thus, there is still no consensus on the relationship between *H. pylori* infection and periodontal condition. This can be attributed to several methodological differences, such as study design, sample population characteristics, and even regional differences [26].

In our study, there was no significant difference in the history of gastric diseases among those infected with *H. pylori* in their blood compared to those who were not infected. According to a report from a large cohort study of approximately 110,000 residents, those with the history of *H. pylori* infection had a significantly higher incidence of gastric disease than those without the history of gastric disease [28]. This may be due to the fact that the participants in our study are young in age. The preferred age for gastric cancer and gastric ulcer is 50 years or older, and the incidence increases over time [29]. We plan to continue to investigate the incidence of gastric diseases in *H. pylori* infection in blood over time.

Commented [HYE3]: I think you may try to explain why H pylori infection was more prevalent in patients with regular dental checkups. This finding is worth noting.

Our present study has some limitations. First, since this was a cross-sectional study, the timing of caries incidence was not confirmed. In the future, we should investigate the effects of period without caries treatment and severity of dental caries on *H. pylori* infection in blood. In addition, there is a possibility that false-positive participants in our antibody test were included. In the future, confirmation by urea breath test and gastric endoscopy will be considered for those who are positive for *H. pylori* infection in blood.

Conclusions

Our study showed that Japanese adults who harbor higher those with decayed teeth have higher risk for *H. pylori* infection in blood. Untreated dental caries may have an impact on systemic *H. pylori* infection.

Acknowledgements

Our study was supported in part by Grant-in-Aid for Young Scientists (20K18809) from the Ministry of Education, Culture, Sports, Science and Technology of Japan.

Conflict of Interest None

to declare.

Author contributions

Our study was carried out in collaboration between all authors. Komei Iwai, Tetsuji Azuma, and Takaaki Tomofuji conceived the study. Takatoshi Yonenaga, Kazutoshi

Watanabe, Akihiro Obora, Fumiko Deguchi, Takao Kojima, and Takaaki Tomofuji collected the samples. Komei Iwai and Takaaki Tomofuji analyzed the data, interpreted the results, and wrote the manuscript. All authors approved the final manuscript.

Orcid

https://orcid.org/0000-0001-5608-743

References

1.Marshall B, Warren JR (1984) Unidentified curved bacilli in the stomach of patients with gastritis and peptic ulceration. The Lancet 323(8390): 1311–1315.

2.IARC Working Group on the Evaluation of Carcinogenic Risk to Humans (1994) Schistosomes, Liver Flukes and *Helicobacter pylori* IARC Monographs on the Evaluation of Carcinogenic Risks to Humans Lyon (FR). IARC 61: 177–241.

3.Correa P, Haenszel W, Cuello C, Tannenbaum S, Archer M (1975) A model for gastric cancer epidemiology. The Lancet 306(7924): 58–60.

4.World Health Organization (2012) Biological agents, a review of human carcinogens; IARC monographs on the evaluation of carcinogenic risks to humans. International Agency for Research on Cancer 100: 1–441.

5.Plummer M, Martel C, Vignat J, Ferlay J, Bray F, Franceschi S (2016) Global burden of cancers attributable to infections in 2012: a synthetic analysis. Lancet Glob Health 4(9): 609–616.

6.Hirayama Y, Kawai T, Otaki J, Kawakami K, Harada Y (2014) Prevalence of *Helicobacter pylori* infection with healthy subjects in Japan. Journal of Gastroenterology and Hepatology 29: 16–19.

7.Ministry of Health, Labour and Welfare (2021) Patient Survey. World Wide Website. Available: https://www.mhlw.go.jp/toukei/list/10-20.html. Accessed 2021 Aug 31.

8.Amiri N, Abiri R, Eyvazi M, Zolfaghari MR, Alvandi A (2015) The frequency of *Helicobacter pylori* in dental plaque is possibly underestimated. Archives of Oral Biology 60(5): 782–788.

9.Bago I, Bago J, Plečko V, Aurer A, Majstorović K, Budimir A (2011) The effectiveness of systemic eradication therapy against oral *Helicobacter pylori*. Journal of Oral Pathology & Medicine 40(5): 428–432.

10.Ogaya Y, Nomura R, Watanabe Y, Nakano K (2015) Detection of *Helicobacter pylori* DNA in inflamed dental pulp specimens from Japanese children and adolescents. Journal of Medical Microbiology 64(1): 117–123.

11.Iwai K, Watanabe I, Yamamoto T, Kuriyama N, Matsui D, Nomura R, Ogaya Y, Oseko F, Adachi K, Takizawa S, Ozaki E, Koyama T, Nakano K, Kanamura N, Uehara R, Watanabe Y (2020) Association between *Helicobacter pylori* infection and dental pulp reservoirs in Japanese adults. BMC Oral Health 19(1): 267.

12.Nomura R, Ogaya Y, Matayoshi S, Morita Y, Nakano K (2018) Molecular and clinical analyses of *Helicobacter pylori* colonization in inflamed dental pulp. BMC Oral Health, 18(1): 64.

13.Rodopi E, Evangelos P, Christina P (2021) Impact of Epigenetic Alterations in the Development of Oral Diseases. Bentham Science 28(6): 1091-1103.

14.Hans Z (2011) Dental infection and vascular disease. Semin Thromb Hemost 37(3): 181-192.

15.Diaconu S, Predescu A, Moldoveanu A, Pop C, Fierbințeanu-Braticevici C (2017) Helicobacter pylori infection: old and new. Journal of Medicine and Life 10(2): 112-117.

16.Kazunari M, Tomoari K, Hiroki I, Hiroshi I, Hiroshi M, Minoru F, Kenichi T, Akiko S, Kazuhiro M, Seiji S, Tadayoshi O, Masaaki K, Ayako A, Tetsuya O, Atsunari N, Jiro H, Ken H, Toshio F (2011) An evaluation of the performance of a novel stick-type kit for rapid detection of Helicobacter pylori antibodies in urine. Clinical Laboratory 57: 481487.

17.Jo F (2018) Caries Epidemiology and Its Challenges. Monogr Oral Science 27: 11-23.

18.World Health Organization (2013) Oral health surveys: basic methods - 5th edition.

19.Sultan Q, Shereen A, Estabraq M, Jood S, Abdelfattah AT, Adil A (2019) Electronic cigarette among health science students in Saudi Arabia. Ann Thorac Med 14(1): 56-62.

20.John DC, Mark BR, Brandi M, Maria CG, Danielle R (2014) Drinking behavior among low-income older adults: a multimethod approach to estimating alcohol use. Alcohol Clin Exp Research 38(11): 2862-2868.

21. Patrick AF, Philip R, Helen VW, Janet EC, Dwayne B, Paul VB (2020) Recall intervals for oral health in primary care patients. Cochrane Database of Systematic Reviews 14;10(10).

22.Ying C, Guili D, Dandan W, Jingyi Y, Yixin Z, James AG, Yihua C, Yixiang W, Bin X (2021) Comparative Microbial Profiles of Caries and Black Extrinsic Tooth Stain in Primary Dentition. Caries Research 55: 310–321.

23.Hamada M, Nomura R, Ogaya Y, Matayoshi S, Kadota T, Morita Y, Uzawa N, Nakano K (2019) Potential involvement of *Helicobacter pylori* from oral specimens in overweight body-mass index. Scientific reports 9(1): 4845.

24.Hisham B, Thenmozhi V, Amirtharaj F, Rola A, Saaid AS (2020) Dental Caries-A Hiding Niche for *Helicobacter Pylori* in Children. J Clin Pediatr Dent 44(2): 90-94.

25.Chaochen W, Nishiyama T, Kikuchi S, Inoue M, Sawada N, Tsugane S, Yingsong L (2017) Changing trends in the prevalence of *H. pylori* infection in Japan (1908-2003): a systematic review and meta-regression analysis of 170,752 individuals. Scientific reports

7(1): 15491.

26.Asqah M, Hamoudi N, Anil S, Jebreen A, Hamoudi WK (2009) Is the presence of *Helicobacter pylori* in dental plaque of patients with chronic periodontitis a risk factor for gastric infection?. Can J Gastroenterol 23: 177–179.

27.Yanqing L, Rong L, Xiaomeng X, Tong X, Yuxiao L, Qin D, Junchao L, Jingbo L, Yaping P, Dongmei Z (2020) eriodontal disease and *Helicobacter pylori* infection in oral cavity: a meta-analysis of 2727 participants mainly based on Asian studies. Clinical Oral Investigate 24(7): 2175-2188.

28.Yatsuya H, Toyoshima H, Tamakoshi A, Kikuchi S, Tamakoshi K, Kondo T, Mizoue T, Tokui N, Hoshiyama Y, Sakata K, Hayakawa N, Yoshimura T (2004) Individual and joint impact of family history and *Helicobacter pylori* infection on the risk of stomach cancer: a nested case–control study. British Journal of Cancer 91(5): 929–934.

29.Talley NJ, Axon A, Bytzer P, Holtmann G, Lam SK, Zanten SV (1999) Management of uninvestigated and functional dyspepsia: a Working Party report for the World Congresses of Gastroenterology 1998. Alimentary Pharmacology & Therapeutics 13(9): 1135-1148.

Table

Table 1. Characteristics of the study participants with and without *H. pylori* infection in blood.

Factor	*H. pylori* infection in blood		*p* value[*]
	Absence (n = 669)	Presence (n = 83)	
Age (years)			
-49	236 (35%)	35 (42%)	
50-59	230 (34%)	30 (36%)	0.256
60-	203 (31%)	28 (22%)	
Male [a]	453 (68%)	60 (72%)	0.398
Smoking habits [b]	74 (11%)	12 (15%)	0.359
Drinking habits [b]	252 (38%)	27 (33%)	0.361
Hypertension [b]	36 (5%)	2 (2%)	0.244
Diabetes [b]	18 (3%)	2 (2%)	0.881
Gastric disease [b]	104 (16%)	9 (11%)	0.258
Heart disease [b]	43 (6%)	3 (4%)	0.313
Sleep disorder [b]	142 (21%)	22 (27%)	0.272
Regular dental checkups [b]	57 (9%)	12 (15%)	0.077
Antibiotic medication history [b]	92 (14%)	6 (7%)	0.096
Periodontal pocket (mm) [b]			
-3	265 (40%)	39 (47%)	0.196
4-	404 (60%)	44 (53%)	
Gingival bleeding [b]	340 (51%)	47 (57%)	0.318
Number of present teeth [b]			
-20	19 (3%)	1 (1%)	
21-23	28 (4%)	4 (5%)	0.644

24-	622 (93%)	78 (94%)	
Decayed teeth [b]	79 (12%)	33 (40%)	< 0.001

Missing teeth [b]	268 (40%)	41 (49%)	0.103
Filled teeth [b]	654 (98%)	82 (99%)	0.537

[*] $p < 0.05$, using the Fishers exact test or the Mann-Whitney *U* test.

[a] Male (percentage of male); [b] presence (percentage of presence).

**Factor**		Crude ORs	95% Cl	*p* value
Age (years)	-49	1	(reference)	0.219
	50-	0.747	0.470 - 1.188	
Gender	Female	1	(reference)	0.399
	Male	0.804	0.484 - 1.335	
Smoking habits	N0	1	(reference)	0.361
	Yes	1.359	0.704 - 2.623	
Drinking habits	N0	1	(reference)	0.361
	Yes	0.798	0.491 - 1.296	
Hypertension	N0	1	(reference)	0.257
	Yes	0.434	0.103 – 1.837	
Diabetes	N0	1	(reference)	0.881
	Yes	0.893	0.203 – 3.919	
Gastric disease	N0	1	(reference)	0.261
	Yes	0.661	0.621 - 1.361	
Heart disease	N0	1	(reference)	0.546
	Yes	0.546	0.166 - 1.800	
Sleep disorder	N0	1	(reference)	0.273
	Yes	1.338	0.795 - 2.255	
Regular dental checkups	N0	1	(reference)	0.081
	Yes	1.815	0.929 - 3.544	
Antibiotic medication history	N0	1	(reference)	0.102
	Yes	0.489	0.207 - 1.154	
Periodontal pocket (mm)	-3	1	(reference)	0.198
	4-	0.740	0.468 - 1.170	

Gingival bleeding	N0	1	(reference)	0.319
	Yes	1.263	0.798 - 2.001	
Number of present teeth	-23	1	(reference)	0.735
	24-	1.179	0.455 - 3.053	
Decayed teeth	N0	1	(reference)	< 0.001

Table 2. Crude odds ratios and 95% CI for *H. pylori* infection in blood.

	Yes	4.929	2.995 - 8.114	
Missing teeth	N0	1	(reference)	0.104
	Yes	1.461	0.925 - 2.307	
Filled teeth	N0	1	(reference)	0.543
	Yes	1.881	0.245 - 14.424	

Abbreviations: ORs, odds ratios; CI, confidence interval.

Table 3. Adjusted odds ratios and 95% CI for *H. pylori* infection in blood.

**Factor**		Adjusted ORs	95% CI	*p* value
**Model 1**				
Age	-49	1	(reference)	0.177
	50-	0.725	0.454 - 1.157	
Gender	Female	1	(reference)	0.318
	Male	0.770	0.462 - 1.285	
Gastric disease	N0	1	(reference)	0.307
	Yes	0.683	0.328 - 1.421	
Regular dental checkups	N0	1	(reference)	0.056
	Yes	1.939	0.982 - 3.830	
Antibiotic medication history	N0	1	(reference)	0.121
	Yes	0.504	0.212 - 1.198	
Decayed teeth	N0	1	(reference)	< 0.001
	Yes	5.017	3.031 - 8.305	
**Model 2**				
Age	-49	1	(reference)	0.095
	50-	0.656	0.401 - 1.076	
Gender	Female	1	(reference)	0.578
	Male	0.860	0.506 - 1.462	
Gastric disease	N0	1	(reference)	0.267
	Yes	2.137	0.551 - 8.566	
Regular dental checkups	N0	1	(reference)	0.414
	Yes	1.359	0.651 - 2.835	
Antibiotic medication history	N0	1	(reference)	0.065
	Yes	0.226	0.046 - 1.097	

Decayed teeth	N0	1	(reference)	< 0.001
	Yes	5.656	3.374 - 9.479	

Abbreviations: ORs, odds ratios; CI, confidence interval.

Model 1: Adjustment for age and gender.

Model 2: Adjustment for age, gender, gastric disease, regular dental checkups, antibiotic medication history, and decayed teeth.

Table 4. Differences in prevalence rate of *H. pylori* infection in blood according to different number of decayed teeth.

Factor	Number of decayed teeth				p value
	0 (n = 640)	1 (n = 66)	2 (n = 30)	3- (n = 16)	
H. pylori infection in blood	50 (8%)	13 (20%)	10 (33%)	10 (63%)	< 0.001

using the chi-square test.
