# Supplementary material for: Association between dental caries and Helicobacter pylori infection in Japanese adults: A cross-sectional study
Source: PLoS One. 2022 Jul 14;17(7):e0271459. doi: 10.1371/journal.pone.0271459 (PMC9282579; doi:10.1371/journal.pone.0271459)
Supplement: Attachment — Submitted filename: Our responses to the reviewers 20220406.docx [file pone.0271459.s002.docx]

**Response to Reviewers**

We greatly appreciate important comments by editor and reviewers gave us. We have introduced revisions to the text in accordance with reviewer’s comments, which we believe have considerably improved it. We have highlighted the changes to our manuscript using blue font.

PONE-D-21-36232

Title: Association between dental caries and *Helicobacter pylori* infection in Japanese adults: A cross-sectional study

Reviewers’ Comments:

Response to Reviewer 1

Please find my little remarks attached to the PDF; Do both tests have the same degree of sensitivity? Please elaborate on this.

**Response: We thank the reviewer for this valuable recommendation. We have added the sentences regarding details on sensitivity and specificity of *Helicobacter pylori* (*H. pylori*) antibody test and pepsinogen test (lines 80-82).**

Please find my little remarks attached to the PDF; In your study, you used the number of decayed and missing teeth which was a quantitative enumeration that does not reflect the severity of dental caries. You may hint to that in the discussion part.

**Response: We thank the reviewer for this excellent suggestion. As you indicated, our study did not investigate dental caries severity. Since the size of caries cavity could have effects on the relationship between dental caries and systemic *H. pylori* infection, the evaluation of dental caries severity may be important to improve the reliability of our observations. This limitation has emphasized in the Discussion section (lines 198-202).**

Please add to the discussion part a justification why patients who experience regular dental checks had more tendency to be infected with *H. pylori*.

**Response: We thank the reviewer for this valuable recommendation. We have added the sentences (line 190-194).**

You may also recommend the use of ICDAS caries scoring to study the severity of caries in relation to *H. pylori* infection even though I understand that this was a retrospective study.

**Response: We thank the reviewer for this excellent suggestion. We did not use ICDAS caries scoring in the present study. However, we will use ICDAS caries scoring in our next research.**

Response to Reviewer 2

1. Reduce your introduction.

**Response: We thank the reviewer for this valuable recommendation. We have reduced introduction.**

2. Please mention how you arrive at the sample size.

**Response: Thank you for your valuable advice. Our study included all participants who had undergone both *H. pylori* testing (*H. pylori* antibody test and pepsinogen test) and dental checkups at Asahi University Hospital Human Health Center between April 2018 and March 2019 (lines 68-70). Therefore, we did not calculate the sample size.**

3. mention which sampling strategy you have adopted, whether universal or purposive.

**Response: We thank the reviewer for this valuable recommendation. Our study employed a universal sampling technique.**

4. Please add the public health significance in conclusion section.

**Response: We thank the reviewer for this valuable recommendation. We have added the sentence (lines 208-210).**

5. Please explain the first sentence of Conclusion, need to check the sentence, grammar section.

**Response: We thank the reviewer for this valuable recommendation. We have revised the sentence (lines 206-208).**

6. It will be useful if any specific message targets towards practice of family physicians. What a family physician should check for and do to prevent dental caries.

**Response: We thank the reviewer for this valuable recommendation. Some people with dental caries do not undergo dental treatment. If dental caries is present and remains untreated, please encourage the patient to undergo dental treatment. We would also appreciate it if you could encourage them to encourage regular dental checkups to prevent dental caries.**

7. Can you mention why significantly less number of females in comparison to male patients, any specific reason or habit responsible.

**Response: We thank the reviewer for this valuable recommendation. In Japan, the proportion of women receiving health checkups tends to be lower than the proportion of men [Ministry of Health, Labour and Welfare (2019) National Survey of Living Standards. World Wide Website. Available: https://www.mhlw.go.jp/toukei/list/20-21kekka.html. Accessed 2022 Mar 18], which is like our study. The reason why the proportion of women receiving health checkups tends to be lower than the proportion of men may be due to their busy housework and child-rearing schedules, but it is not understood completely.**

We answer in response to the reviewer's comments as noted above. We would like to express our sincere appreciation for your very useful suggestions. We look forward to working with you in the future.
